# Effects of different heavy sled loads sprint training on acceleration performance in adolescent sprinters

**DOI:** 10.1186/s13102-025-01370-5

**Published:** 2025-12-01

**Authors:** Lili Jia, Biyu Zhang, Tseching Liang, Guojie Wang, Miaoyu Han, Xuebing Zhang

**Affiliations:** 1 School of Education, Beijing Sport University, Beijing, 100084 China; 2 Department of Athletics, National Taiwan University, Taipei City, 10617 Taiwan; 3 Nanjing Sport Institute, Nanjing, Jiangsu 210033 China; 4 Facultad de Ciencias de la Actividad Física y del Deporte, Universidad Politécnica de Madrid, Madrid, 28040 Spain

**Keywords:** Resisted sprinting, Sled towing, Sprint acceleration, Step length

## Abstract

**Background:**

Sled sprint training to improve athletes’ sprint performance is becoming increasingly popular. However, there is still no consensus on the appropriate resistance to improve acceleration, and few studies have focused on adolescent sprinters. This study aimed to investigate the effects of 8 weeks of different heavy-load sled sprint training on acceleration performance and kinematics in adolescent sprinters.

**Methods:**

Thirty-two adolescent sprinters were assigned to four groups: three resisted groups (25%, 40%, and 50% body mass [BM]; *n* = 8 each) and one non-resisted group (NRS; *n* = 8). Participants trained twice weekly for 8 weeks. Pre- and post-training assessments included 30 m and 60 m sprint performance and kinematic parameters (step length, step frequency, contact time, flight time, trunk angle, push-off angle, shin angle, and hip angular velocity).

**Results:**

For sprint performance, within-group improvements were significant in 30 m for 40% and 50% BM groups and in 60 m for 25% BM group (*p* < 0.05). Between-group comparisons showed greater 30 m improvements in 40% and 50% BM groups compared to NRS group (*p* < 0.05). In terms of sprint kinematics, within-group improvements were significant for step length (0–30 m) and step frequency (10–20 m) across all resisted groups, and for trunk angle (toe-off) (0–20 m) and push-off angle (10–20 m) in the 40% and 50% BM groups. Between-group comparisons showed significantly greater trunk angle (10–20 m) in 40% and 50% BM groups compared to NRS group (*p* < 0.05).

**Conclusions:**

These results indicate that heavier sled training (40% and 50% BM) was more effective in enhancing speed, trunk angle, and step length in acceleration, whereas lighter sled training (25% BM) benefited 60 m sprint performance. This study highlights how different training loads impact sprint performance, providing coaches with insights into kinematic changes following prolonged training and aiding in the optimization of sprint training programs for adolescents.

**Supplementary Information:**

The online version contains supplementary material available at 10.1186/s13102-025-01370-5.

## Introduction

Speed in the acceleration phase is a key element in determining the overall speed of a sprint and is highly correlated with maximal speed performance, which affects 100 m sprint performance [1]. The effectiveness of acceleration techniques directly affects an athlete’s acceleration distance, maximal speed performance, ability to maintain maximal speed, and speed endurance performance [2]. Training methods for acceleration focus on overcoming inertia, transitioning efficiently from a static state, and effectively controlling body posture to optimize the conversion of ground reaction force (GRF) into propulsive force [3]. In sprints and sports that require frequent changes of direction, such as football and rugby, strong acceleration is a key factor for athletes [4]. Various training methods have been developed to acutely and chronically improve sprint acceleration to improve overall performance [5–7].

The movement pattern, movement rate, and muscle activation in the sled-towing sprint are particularly similar to the technical characteristics of the acceleration phase, such as forward body lean, low center of mass, and high explosive force production [8, 9]. Therefore, this training method is of great research importance in the development of sprint acceleration. Sled loads are typically based on either a percentage of body mass (BM) [10–13] or velocity decrement (VDec) [14–16]. In practice, many studies determine sled towing load as a percentage of the athlete’s body mass, based on the rationale that taller athletes generally produce greater muscle strength output [11, 12, 17, 18]. This approach allows consistent kinematic comparisons across individuals and aligns with practical coaching methodologies [10–14]. For example, Cross et al. [19] conducted acute research that suggested the optimal load to maximize force production in sled sprints is approximately 50% VDec (69%-96% BM). Separately, Kawamori et al. demonstrated that heavier loads (30% BM) acutely increase contact time and horizontal force [18]. Evidence from longitudinal interventions also supports the efficacy of various loading strategies: Zafeiridis et al.‘s protocol using 5 kg sleds demonstrated improved acceleration performance (0–20 m) after resisted training [20], while Spinks et al. reported that an 8-week training program with 12.6% BM loads enhanced acceleration sprint performance (0–15 m), whereas the non-resisted group demonstrated no significant improvement [4].

Current evidence indicates that the appropriate resistance load for developing sprint speed is between 10 and 150% BM, with a single sprint distance of 20–60 m, and that the training adaptations at the same resistance load intensity are different for different training levels and stages [8, 21, 22]. Recent training studies have increasingly focused on heavier resisted loads to improve acceleration [12, 17, 19, 23, 24]. Acute studies demonstrate that sprint kinematic parameters such as contact time, stride length, and stride frequency change significantly with increasing sled load [13, 25, 26]. For instance, training with sled loads of 30% or 75% BM has been shown to improve subsequent sprint kinematics and performance [6, 27]. Evidence further suggests that moderate to heavy sled loads (10%-20% BM to >30% BM) provide an effective overload, enhancing acceleration through more efficient force application, which is a key determinant of sprint acceleration [2, 8, 28, 29]. This approach appears particularly relevant given that propulsive force during acceleration is reported to be 46% greater than at maximum velocity, underscoring the value of overload training for improving propulsive force components of GRF [30, 31]. While heavier loads elicit greater acute neuromuscular stress and alter muscle activation patterns [32], acute studies indicate that very heavy loading (≥ 125% BM) may impair sprint efficiency and induce fatigue [6, 33]. These acute changes may determine long-term adaptations. Despite considerable studies [4, 10–12, 14, 15, 17, 20, 23, 24], no consensus has been reached on the optimal sled load for sprint acceleration training.

In sprint kinematics, speed is determined by the product of stride length and stride frequency [5]. Compared to unresisted sprinting, resistance loading typically results in shorter stride length, lower stride frequency, reduced flight time, and increased contact time [9, 34]. Improvements in sprint performance after resistance training are mainly attributed to increased stride length [5, 14]. Additionally, lower limb kinematics show positive training effects in unloaded conditions, as evidenced by changes in trunk angle, shin angle, and hip extension angle [6, 27, 35]. The extent of these changes is determined by the weights used during the sled training [36]. However, studies investigating the long-term effects of different loaded sled sprints on sprint acceleration are limited and have not demonstrated changes in the kinematics of unloaded sprints after long-term training [5, 10–12, 36, 37]. Although Stavridis et al. [23] demonstrated improved acceleration kinetics in adults using 50% VDec sled loads and training 12 sessions over 6 weeks, the long-term kinematic adaptations to heavy loads in adolescent sprinters remain unclear.

Systematic reviews indicate that resistance sprint training is more effective during the preparation phase and that individuals with lower exercise levels or without prior resistance training experience may experience greater adaptations [8, 21]. Most research has primarily focused on adult athletes, including college athletes [13, 16, 22, 38], adult males and females [14, 39], and elite or recreational athletes in various team sports [4, 24, 25]. Despite extensive studies on sled sprinting, no consensus exists on the optimal resistance for acceleration improvement. Furthermore, most research has focused on adult athletes, with limited data on adolescent sprinters. What kind of changes in acceleration performance and kinematics are induced by the long-term use of heavier sled loads in them? To address this gap, distinct loads were selected: 25% BM (representing a light load aligned with typical coaching recommendations for acceleration training [11, 14]), 50% BM (a heavy exploratory load exceeding conventional thresholds to probe neuromuscular adaptations [12, 19, 23]), and 40% BM (a moderate-heavy load bridging [12]). This study aimed to examine whether these loads alter the kinematic characteristics of the acceleration phase (0–30 m) and improve sprint performance at 30 m and 60 m in adolescent sprinters. It was hypothesized that heavier loaded (40% and 50% BM) sled training would improve 30 m sprint performance and kinematics. The findings will help coaches understand the effects of different loading strategies, enabling them to develop more effective loaded sled training programs for youth sprinters.

## Materials and methods

### Experimental approach to the problem

This study used a randomized, longitudinal experimental design to compare the effects of three sled loads (25%, 40%, and 50% BM) and non-resisted sprint training on acceleration kinematics and performance in adolescent sprinters. BM-based loading was chosen for its high practical utility and ease of implementation for coaches, as well as its accessibility in youth sport settings that typically lack the specialized equipment required for velocity decrement (VDec) prescription. Following a pretest to assess 30 m sprint performance, participants were matched based on their results and allocated into 4 groups: a non-resisted sprint (NRS) group, and three resisted groups training at 25% BM, 40% BM, or 50% BM, with baseline acceleration being similar across groups (Table 1). All groups then completed an 8-week, twice-weekly training intervention. Pre- and post-intervention assessments included 30 and 60 m sprint tests, alongside a kinematic analysis of the 30 m sprint. Two familiarization drills were conducted in the week before the first test, to develop the correct technique for executing resistance sled sprint.

**Table 1:** Age and anthropometric data of the participants

Group(*n* = 8)	Age(y)	Height(cm)	Body mass (kg)	30 m (s)
25%BM	16 ± 1.0	180 ± 2.79	66.22 ± 4.76	4.144 ± 0.038
40%BM	15 ± 0.7	179 ± 4.55	67.81 ± 4.38	4.130 ± 0.062
50%BM	16 ± 1.0	178 ± 6.50	66.90 ± 4.69	4.136 ± 0.071
NRS	16 ± 1.1	180 ± 3.11	66.16 ± 4.83	4.142 ± 0.063

^*^ BM = Body mass; NRS = non-resisted sprint; Data were taken before the 8-week training period and are presented as mean ± SD

### Participants

Thirty-two male adolescent sprinters were recruited for this study (participant characteristics are presented in Table 1). Their performance standards ranged from amateur to regional-level athletes. Participants had at least three years of sprint training experience and had trained five times weekly for six months before the study. They had no injuries or medical conditions affecting their performance. Participants were instructed to maintain a regular diet and training regimen throughout the study. All participants and their parents or guardians were fully informed of the study protocol, potential risks and benefits, and provided written consent. The study was approved by the Institutional Ethics Committee of Beijing Sport University (No. 2023019 H).

### Procedures

#### Testing procedures

The tests and training were implemented during the preseason preparation period. Participants were first weighed to determine the training loads relative to 25, 40, and 50% of their body mass. They then completed a standardized 20-minute warm-up, including jogging, a variety of movement drills, and dynamic stretching exercises, to prepare for maximum-effort sprinting. Following a 5-minute recovery period after the warm-up, they completed 2 maximum-effort sprints of 30 m and 60 m, respectively, with a 3-minute rest between trials. The fastest sprint trial for each participant was used in the statistical analysis. All pre- and post-test sprint trials were conducted on an IAAF-accredited stadium with a Mondo track surface during the same time window (9:00 to 11:00 am). Environmental temperature ranged from 19 to 23 °C, relative humidity was 40–55%, and wind speed was ≤ 2.0 m/s. Electronic timing gates (Brower TC-System, Brower Timing Systems, USA) were positioned at the start line, at 30 m, and at 60 m. To standardize starting positions, all participants adopted a three-point stance with the left foot leading, thereby making the right limb the rear push-off leg. All wore their athletic clothes and spiked sprint shoes. Strong verbal encouragement was provided during testing and training to increase motivation. All procedures were replicated post-training.

#### Kinematic analysis

**Fig. 1:**
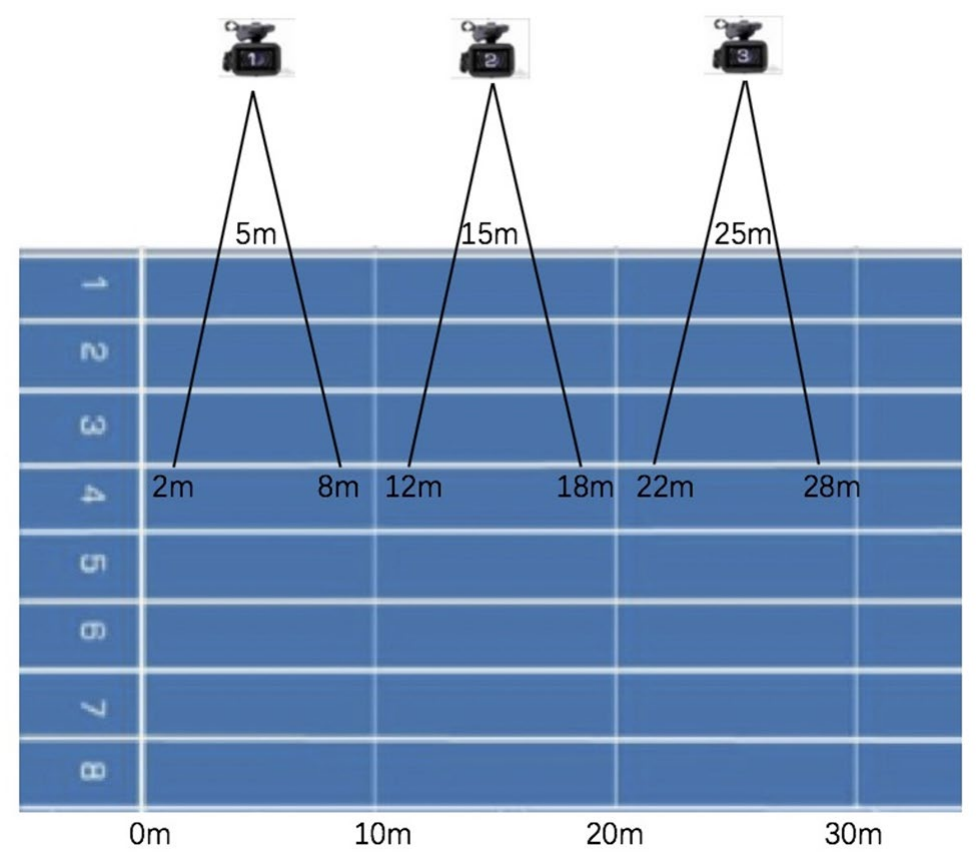
Settings of the kinematics data collection

During the 30 m sprint tests, kinematic parameters were collected using a standard two-dimensional method. Three high-speed video cameras (FDR AX700, Sony, Japan, frame rate 50 Hz, 4 K resolution, 1/1000 s shutter speed) positioned at 5 m, 15 m, and 25 m from the start [30, 34, 36]. Each camera was positioned on the right side of the sprinting lane, 25 m from its proximal edge, aligned perpendicular to the participants’ line of movement, and at a height of 1.20 m. This provided a field of view from the 2–8 m, 12–18 m, and 22–28 m marks of the sprint, respectively. Each camera was calibrated using a 1 m calibration cube, with 4 retro-reflective body markers filmed in the optical plane for 10 s to ensure accurate data acquisition. A comprehensive diagram of the experimental setup is shown in Fig. 1. The first complete stride cycle (2 steps) of the right limb within the camera’s field of view was used for data analysis. The right limb was selected for all participants as it was the consistently instrumented side and functionally served as the primary propulsive limb during the sprint start (as the rear leg in the three-point stance).

The data acquisition video was processed and analyzed using Kinovea (Kinovea 0.9.5, www.kinovea.org), a software package widely used for motion analysis capabilities [40]. Following manual calibration procedures, the software automatically captured reflective marker points; subsequently, all trajectories underwent manual frame-by-frame review to correct errors or missing points, thereby ensuring data accuracy [32, 40, 41]. Specifically, five markers are attached to the right side of the body to assist in the digital sprint kinematics: the acromion process (shoulder), the greater trochanter of the femur (hip), the lateral condyle of the tibia (knee), the lateral malleolus of the fibula (ankle), and the head of the fifth metatarsal (toe, marked on the outer surface of the sprint shoe). The sprint kinematic parameters analyzed in this study are widely used in sprint biomechanics research to quantify technical execution [5, 13, 16, 32, 34, 36, 41]. The instant of touch-down and toe-off were identified as the first video frame in which the foot made contact with the ground and the first frame in which it left the ground, respectively. All joint angles were measured at these key instants. The parameters included:

##### Step length

Horizontal distance between the initial ground contact of one foot to the initial ground contact of the other foot.

##### Step frequency

Reciprocal of step time (contact time + flight time).

##### Contact time

Duration from initial foot contact to toe-off.

##### Flight time

Duration from toe-off of one foot to initial contact of the opposite foot.

##### Trunk angle (touch down/ toe-off)

Angle between the line connecting the shoulder and hip markers and the horizontal plane.

##### Shin angle (touch down)

Angle between the line connecting the knee and ankle markers and the horizontal at touch down.

##### Push-off angle

Angle between the line connecting the knee and ankle markers and the horizontal at toe-off.

##### Hip extension angular velocity

Mean angular velocity in the hip joint of the support leg from touchdown to toe-off.

##### Hip flexion angular velocity

Mean angular velocity in the hip joint of the swing leg from toe-off to moment of the hip maximum flexion.

#### Training program

The sled sprint training protocols were conducted twice weekly for the 8 weeks on Tuesdays and Thursdays. In addition to the experimental sled sprint training, all participants completed an identical periodized training program 3 days per week (on non-sled sprint training days) for the duration of the study as part of their regular training program. During the study period, participants were instructed to abstain from any additional resistance or sprint-specific training outside the prescribed experimental and periodized training programs.

Each training session began with a 30-minute warm-up during which all participants performed dynamic stretching, sprint-specific neuromuscular coordination exercises, and various footwork and agility exercises. After the warm-up, participants in all 4 groups completed the sprint training protocol listed in Table 2. This sprint training protocol was roughly based on the volume and frequency outlined in previous sprint training studies [8, 9, 17, 42]. The 25%, 40%, and 50%BM groups completed all sprints with resistance, except for the last 3 60 m sprints of each session (to reinforce proper sprinting technique when not resisted), whereas the NRS group completed all sprints without resistance. The resistance groups each pulled a sled with weighted plates loaded on top of the sled. The weight of the sled used was 16.5 kg, and the drawstring (length:2.7 m) tied around the participant’s waist. Participants wore athletic clothing and spiked shoes during each training session.

**Table 2:** Training program

Week	Sessions	Content	[Distance(m)×repetition] × sets	Intensity (%)	Rest
1–8	2/16	Sled / Sprint towing sprint	[30 × 6]x3	100%	rep:2 min set:3 min
		Sprint training	60 × 3		

A certified strength and conditioning specialist supervised all training sessions, ensuring that all warm-up activities and sprints were performed with the correct technique and maximum effort. After completion of the training protocol, participants were required to rest for 1 day. Post-test measurements were performed identically to the pre-test procedures described above.

### Statistical analyses

Descriptive statistics are presented as mean ± SD. The data passed the normality test (Shapiro-Wilk) and the homogeneity of variance test (Levene), which met the analysis requirements. A one-way ANOVA was performed on the baseline measures of 30-m sprint time to determine whether the four groups were evenly matched in terms of sprint performance before the training intervention. Within-group comparisons were made using paired t-tests to identify significant differences between pre-tests and post-tests for each variable. The magnitude of within-group effects was quantified using Cohen’s d, interpreted as follows: < 0.50 = small; 0.50–0.8 = moderate; and > 0.80 = large. For variables that showed significant within-group changes, a one-way ANOVA was performed on post-test results to compare differences among the four groups. For any variable where a significant main effect was identified, effect size was reported as eta-squared (η²), with values of 0.01, 0.06, and 0.15 representing small, medium, and large effects, respectively. And further analysis was conducted by LSD test post hoc and between-group Cohen’s d values were computed to further clarify pairwise comparisons. The alpha level was set at *p* < 0.05. All statistical analyses were performed using SPSS (Version 22.0; SPSS Inc., Chicago, IL).

## Results

### Sprint performance at 30 m and 60 m

Sprint performance data are presented in Table 3. Following the training intervention, paired sample t-test revealed significant improvements in 30 m sprint time in the 40%BM (-1.07%, *p* < 0.05, ES = large) and 50% BM (-1.26%, *p* < 0.05, ES = large) groups, while the 25% BM group showed a significant improvement in 60 m sprint time (-0.75%, *p* < 0.05, ES = large). A one-way ANOVA results indicated statistically significant differences with a large effect size among groups in 30 m sprint performance (*p* < 0.05, η²=0.29). Post hoc analysis revealed that the 40% BM (–1.07% vs. − 0.10%, ES = large) and 50% BM (–1.26% vs. − 0.10%, ES = large) groups demonstrated significantly greater improvements compared to the NRS group.

**Table 3:** Effect of 8-weeks of training on sprint time at 30 and 60 m

	30 m(s)				60 m(s)			
	Pre	Post	%change	Cohen’s d	Pre	Post	%change	Cohen’s d
25%BM	4.144 ± 0.038	4.120 ± 0.041	-0.58	0.82	7.286 ± 0.065	7.231 ± 0.051^*^	-0.75	1.06
40%BM	4.130 ± 0.062	4.086 ± 0.040^*,a^	-1.07	1.33	7.290 ± 0.131	7.228 ± 0.108	-0.85	0.96
50%BM	4.136 ± 0.071	4.084 ± 0.046^*,b^	-1.26	1.22	7.272 ± 0.059	7.232 ± 0.038	-0.55	0.93
NRS	4.142 ± 0.063	4.138 ± 0.022	-0.10	0.17	7.274 ± 0.076	7.236 ± 0.060	-0.55	0.76

^*^Significant (*P* < 0.05) different pre-vs. post-test; ^a^ Significant (*P* < 0.05, Cohen’s = 1.61) difference between 40%BM group and NRS group; ^b^ Significant (*P* < 0.05, Cohen’s = 1.49) difference between 50%BM group and NRS group

### Kinematic parameters

Stride cycle kinematic data are listed in Table 4. Paired t-tests showed significant increases in step length at 5 m (25%BM: 3.03%; 40%BM: 2.96%; 50%BM: 3.85%; *p* < 0.05, ES = large), 15 m (25%BM: 1.62%, *p* < 0.05; 40%BM: 3.19%, *p* < 0.01; 50%BM: 2.69%, *p* < 0.05; ES = large), and 25 m (25%BM: 3.02%; 40%BM: 4.45%; 50%BM: 2.49%; *p* < 0.05, ES = large) in all resisted groups. Step frequency also improved significantly at 15 m in these groups (25%BM: 6.92%, *p* < 0.01; 40%BM: 7.32%, *p* < 0.01; 50%BM: 3.65%, *p* < 0.05; ES = large). Flight time decreased significantly at 15 m in the 25%BM (-10.94%, *p* < 0.05, ES = large) and 40% BM (-10.77%, *p* < 0.01, ES = large) groups. No significant changes in contact time were observed in any group (*p* > 0.05).

**Table 4:** Effect of 8-weeks of training on kinematic parameters

		Step length (m)				Step frequency (Hz)				Support time (s)				Flight time (s)			
		Pre	Post	%change	Cohen’s d	Pre	Post	%change	Cohen’s d	Pre	Post	%change	Cohen’s d	Pre	Post	%change	Cohen’s d
5 m	25%BM	1.32 ± 0.09	1.36 ± 0.07*	3.03	1.33	4.27 ± 0.46	4.41 ± 0.31	3.28	0.80	0.140 ± 0.010	0.136 ± 0.009	2.86	0.57	0.096 ± 0.017	0.092 ± 0.008	-4.17	0.28
	40%BM	1.35 ± 0.08	1.39 ± 0.06*	2.96	1.13	4.25 ± 0.26	4.39 ± 0.16	3.29	0.50	0.136 ± 0.009	0.132 ± 0.004	2.94	0.57	0.100 ± 0.016	0.096 ± 0.011	-4.00	0.28
	50%BM	1.30 ± 0.05	1.35 ± 0.05*	3.85	1.48	4.53 ± 0.37	4.70 ± 0.35	3.75	0.51	0.132 ± 0.011	0.128 ± 0.015	-3.03	0.57	0.090 ± 0.014	0.086 ± 0.018	-4.44	0.21
	NRS	1.31 ± 0.08	1.34 ± 0.07	2.29	0.67	4.41 ± 0.31	4.36 ± 0.19	-1.13	0.24	0.138 ± 0.008	0.140 ± 0.014	1.45	0.12	0.090 ± 0.020	0.090 ± 0.017	0.00	0.00
15 m	25%BM	1.85 ± 0.09	1.88 ± 0.11*	1.62	1.15	4.19 ± 0.34	4.48 ± 0.22^#^	6.92	1.86	0.112 ± 0.008	0.112 ± 0.008	0.00	0.00	0.128 ± 0.013	0.114 ± 0.005*	-10.94	1.23
	40%BM	1.88 ± 0.08	1.94 ± 0.08^#^	3.19	1.79	4.10 ± 0.16	4.40 ± 0.22^#^	7.32	1.96	0.114 ± 0.009	0.112 ± 0.008	-1.75	0.19	0.130 ± 0.007	0.116 ± 0.005^#^	-10.77	1.66
	50%BM	1.86 ± 0.07	1.91 ± 0.09*	2.69	1.72	4.38 ± 0.42	4.54 ± 0.47*	3.65	1.13	0.110 ± 0.007	0.108 ± 0.008	-1.82	0.19	0.120 ± 0.020	0.114 ± 0.018	-5.00	0.53
	NRS	1.85 ± 0.07	1.87 ± 0.08	1.08	0.76	4.28 ± 0.21	4.35 ± 0.13	1.64	0.20	0.110 ± 0.007	0.108 ± 0.008	-1.82	0.19	0.122 ± 0.008	0.122 ± 0.004	0.00	0.00
25 m	25%BM	1.99 ± 0.08	2.05 ± 0.11*	3.02	1.28	4.14 ± 0.24	4.29 ± 0.25	3.62	0.72	0.110 ± 0.007	0.104 ± 0.005	-5.45	0.53	0.132 ± 0.016	0.130 ± 0.014	-1.52	0.35
	40%BM	2.02 ± 0.10	2.11 ± 0.09*	4.45	1.15	4.19 ± 0.33	4.23 ± 0.35	0.95	0.22	0.106 ± 0.005	0.108 ± 0.008	1.89	0.35	0.134 ± 0.013	0.130 ± 0.012	-2.99	0.57
	50%BM	2.01 ± 0.11	2.06 ± 0.12*	2.49	1.43	4.19 ± 0.33	4.23 ± 0.35	0.95	0.50	0.106 ± 0.005	0.108 ± 0.008	1.89	0.35	0.134 ± 0.013	0.130 ± 0.012	-2.99	0.28
	NRS	2.01 ± 0.06	2.06 ± 0.06	2.49	0.68	4.40 ± 0.22	4.55 ± 0.21	3.41	0.57	0.102 ± 0.008	0.098 ± 0.008	-3.92	0.57	0.126 ± 0.005	0.122 ± 0.004	-3.17	0.47

^*^Significant (*P* < 0.05) different pre-vs. post-test; ^#^Significant (*P* < 0.01) different pre-vs. post-test

Lower limb kinematic data are listed in Table 5. For trunk angle (toe-off), a significant decrease (indicating greater forward lean) was observed in the 40% (-4.90%, ES = large) and 50% BM (-11.98%, ES = large) groups at 5 m (*p* < 0.05), in all resisted groups at 15 m (25%BM: -9.28%; 40%BM: -6.99%; 50%BM: -10.03%; *p* < 0.05,ES = large), and in the 50% BM group (-4.56%, *p* < 0.01, ES = large) at 25 m. The push-off angle decreased significantly in the 40%BM (-2.82%, ES = large), 50%BM (-10.79%, ES = large), and NRS (-6.25%, ES = large) groups at 15 m (*p* < 0.05), and in the 25%BM group (-7.58%, *p* < 0.01 ES = large) at 25 m.

**Table 5:** Effect of 8-weeks of training on kinematic parameters

				5 m	15 m	25 m
Trunk angle (Toe-off)	25%BM	Pre		53.60 ± 4.39	80.04 ± 3.87	84.66 ± 3.65
		Post		48.02 ± 6.63	72.61 ± 2.19*	80.61 ± 3.36
		%change		-10.41	-9.28	-4.78
		Cohen’s d		0.78	1.31	0.75
	40%BM	Pre		53.04 ± 4.69	75.07 ± 3.39	80.64 ± 4.22
		Post		50.44 ± 4.56*	69.82 ± 3.27*^a^	77.43 ± 3.85
		%change		-4.90	-6.99	-3.98
		Cohen’s d		1.13	1.25	0.74
	50%BM	Pre		51.42 ± 3.36	77.84 ± 8.01	82.22 ± 6.14
		Post		45.26 ± 4.60*	70.03 ± 3.08*^b^	78.47 ± 6.69^#^
		%change		-11.98	-10.03	-4.56
		Cohen’s d		0.95	1.05	1.50
	NRS	Pre		52.61 ± 8.71	76.85 ± 8.11	84.07 ± 5.92
		Post		51.87 ± 6.72	75.11 ± 4.53	82.31 ± 3.46
		%change		-1.41	-2.26	-2.09
		Cohen’s d		0.12	0.16	0.37
Push-off angle	25%BM	Pre		39.62 ± 1.52	40.22 ± 1.10	41.41 ± 2.97
		Post		37.89 ± 3.03	38.27 ± 1.64	38.27 ± 1.92^#^
		%change		-4.37	-4.85	-7.58
		Cohen’s d		0.42	0.67	1.58
	40%BM	Pre		40.60 ± 3.36	40.01 ± 2.24	42.60 ± 3.21
		Post		39.02 ± 4.85	38.88 ± 2.39*	39.40 ± 4.28
		%change		-3.89	-2.82	-7.51
		Cohen’s d		0.33	1.13	0.94
	50%BM	Pre		40.87 ± 4.27	42.62 ± 4.39	41.04 ± 3.67
		Post		37.23 ± 4.09	38.02 ± 2.55*	40.64 ± 2.79
		%change		-8.91	-10.79	-0.97
		Cohen’s d		0.42	1.25	0.28
	NRS	Pre		37.03 ± 5.83	41.42 ± 5.73	39.28 ± 4.15
		Post		37.85 ± 4.76	38.83 ± 4.15*	39.43 ± 2.88
		%change		2.21	-6.25	0.38
		Cohen’s d		0.19	0.87	0.06
Trunk angle (Touchdown)	25%BM	Pre		55.07 ± 5.96	78.42 ± 4.72	81.82 ± 5.02
		Post		51.82 ± 4.32	70.66 ± 3.13*	80.67 ± 4.45
		%change		-5.90	-9.90	-1.41
		Cohen’s d		0.51	1.19	0.38
	40%BM	Pre		56.65 ± 5.22	75.61 ± 6.50	76.84 ± 6.69
		Post		51.63 ± 4.39*	70.68 ± 5.32	75.44 ± 4.28
		%change		-8.86	-6.52	-1.82
		Cohen’s d		1.16	0.77	0.22
	50%BM	Pre		55.83 ± 4.38	76.84 ± 7.01	80.85 ± 3.70
		Post		51.06 ± 3.46*	70.87 ± 4.44	78.66 ± 2.30*
		%change		-8.54	-7.77	-2.71
		Cohen’s d		1.06	0.75	1.05
	NRS	Pre		59.64 ± 7.13	75.81 ± 5.89	77.84 ± 6.87
		Post		56.83 ± 7.85	73.42 ± 4.62*	80.22 ± 4.09
		%change		-4.71	-3.15	3.06
		Cohen’s d		0.55	0.39	0.50
Shin angle (Touchdown)	25%BM	Pre	71.26 ± 4.82		85.44 ± 2.07	92.63 ± 2.07
		Post	66.61 ± 4.67*		83.01 ± 3.32	89.80 ± 2.59
		%change	-6.52		-2.84	-3.05
		Cohen’s d	0.99		0.91	0.69
	40%BM	Pre	65.83 ± 3.11		84.27 ± 2.77	89.61 ± 1.82
		Post	63.65 ± 0.55		80.20 ± 1.92	88.07 ± 4.06
		%change	-3.31		-4.83	-1.72
		Cohen’s d	0.52		0.93	0.40
	50%BM	Pre	66.87 ± 4.09		85.40 ± 3.51	90.41 ± 5.59
		Post	65.20 ± 2.86		81.04 ± 3.54	88.20 ± 4.27*
		%change	-2.50		-5.11	-2.44
		Cohen’s d	0.47		0.84	1.06
	NRS	Pre	68.23 ± 4.02		85.82 ± 2.39	90.02 ± 2.35
		Post	67.86 ± 1.92		86.03 ± 7.38	87.82 ± 3.27
		%change	-0.54		0.24	-2.44
		Cohen’s d	0.11		0.02	0.73
Hip extension angular velocity	25%BM	Pre	424.73 ± 28.48		492.36 ± 64.04	486.46 ± 53.50
		Post	425.83 ± 10.86		503.24 ± 73.28	509.56 ± 35.03
		%change	0.26		2.21	4.75
		Cohen’s d	0.03		0.44	0.44
	40%BM	Pre	472.69 ± 89.16		522.10 ± 61.01	516.29 ± 34.39
		Post	488.09 ± 88.38		532.39 ± 74.45	539.94 ± 21.12
		%change	3.26		1.97	4.58
		Cohen’s d	0.13		0.39	0.66
	50%BM	Pre	427.31 ± 60.06		476.92 ± 30.57	481.45 ± 53.31
		Post	449.61 ± 33.73		510.74 ± 24.95*	510.92 ± 41.61
		%change	5.22		7.09	6.12
		ES	0.376		1.14	0.55
	NRS	Pre	452.86 ± 60.31		471.06 ± 39.09	519.74 ± 81.87
		Post	462.16 ± 80.22		512.21 ± 27.31	510.26 ± 80.74
		%change	2.05		8.73	-1.82
		Cohen’s d	0.28		0.71	0.08
Hip flexion angular velocity	25%BM	Pre	407.24 ± 79.84		498.24 ± 153.42	570.82 ± 82.56
		Post	478.36 ± 97.24		569.58 ± 130.53	545.09 ± 77.08
		%change	17.47		14.32	-4.51
		Cohen’s d	0.49		0.37	0.26
	40%BM	Pre	549.99 ± 28.76		569.06 ± 37.40	573.27 ± 93.25
		Post	536.15 ± 93.88		564.27 ± 87.17	589.97 ± 62.51
		%change	-2.53		-0.84	2.92
		Cohen’s d	0.12		0.05	0.03
	50%BM	Pre	541.61 ± 52.36		627.97 ± 111.44	615.09 ± 66.38
		Post	577.33 ± 81.35		631.18 ± 86.56	651.53 ± 29.23
		%change	6.60		0.51	5.92
		Cohen’s d	0.30		0.04	0.42
	NRS	Pre	546.45 ± 66.98		680.27 ± 129.28	556.16 ± 104.51
		Post	568.51 ± 60.31		629.67 ± 64.81	603.40 ± 74.86
		%change	4.04		-7.44	8.50
		Cohen’s d	0.34		0.30	0.77

*Significant (*P* < 0.05) different pre-vs. post-test; ^#^Significant (*P* < 0.01) different pre-vs. post-test; ^a^ Significant (*P* < 0.05, Cohen’s = 1.33) difference between 40%BM group and NRS group; ^b^ Significant (*P* < 0.05, Cohen’s = 1.30) difference between 50%BM group and NRS group

Trunk angle (touch down) decreased in the 40%BM (-8.86%, ES = large) and 50% BM (-8.54%, ES = large) groups at 5 m, in the 25%BM group (-9.90%, ES = large) at 15 m, and in the 50% BM group (-2.71%, ES = large) at 25 m (*p* < 0.05). Shin angle (touch down) decreased in the 25% BM group (-6.52%, ES = large) at 5 m and in the 50% BM group (-2.44%, ES = large) at 25 m (*p* < 0.05). Hip extension angular velocity increased significantly in the 50% BM group at 15 m (7.09%, *p* < 0.05, ES = large), while no significant changes in other groups. No significant differences were found in hip flexion angular velocity within each group (*p* > 0.05).

Following paired t-tests, four parameters with significant improvements-step length, step frequency, trunk angle (toe-off), and push-off angle were selected for one-way ANOVA. The ANOVA revealed a statistically significant difference with a large effect size among groups for trunk angle (toe-off) (*p* < 0.05, η² = 0.32). Post hoc analysis indicated that the 40% BM (–6.99% vs. − 2.26%, ES = large) and 50% BM (–10.03% vs. − 2.26%, ES = large) groups showed significantly greater reductions than the NRS group at 15 m (*p* < 0.05). No significant inter-group differences were found in step length, step frequency, or push-off angle after training (*p* > 0.05) (Table 5).

## Discussion

This study aimed to examine the effects of three different heavy sled loads (25%, 40%, and 50% BM) on the 30 m and 60 m sprint performance and kinematic parameters in acceleration in adolescent sprinters. The main findings were as follows: (a) the 40%BM and 50%BM groups significantly improved 30 m sprint performance compared to the 25% BM and NRS groups, while the 25% BM group enhanced 60 m sprint performance; (b) all resisted groups showed greater improvements in step length than step frequency, with significant increases in step length during the 0–30 m; and (c) the trunk angle (toe-off) and push-off angle of the 40% and 50% BM groups improved during the early acceleration phase.

It is noteworthy that the three loading sled sprint training programs had distinct effects on sprint performance. Specifically, the 40% and 50% BM groups showed significant improvements in 30 m sprint performance (1.07% and 1.26%, respectively), while the 25% BM group showed significant improvements in 60 m sprint performance (0.75%). These results are consistent with previous research indicating that heavy loads enhance acceleration performance, whereas lighter loads improve maximal speed [12, 15, 31, 43–45]. For instance, Morin et al. demonstrated that heavy-loaded sled training (80% BM, 8 weeks, twice weekly) enhances acceleration performance (0–20 m) without affecting maximal speed in adult athletes [29]. Lower-level athletes, such as the adolescent sprinters in our study, may enter the maximal speed phase earlier [46, 47]. Consistent with this, our study found that 25% BM resisted loads had a lesser impact on sprinting technique and did not significantly improve contact time, but they increased step length (1.62%-3.03%) while maintaining constant time, thereby enhancing 60 m sprint performance. This aligns with previous reports that light loads can moderately improve maximal speed without altering sprint mechanics [8, 48]. Although the 25%BM group showed a 0.75% improvement in 60 m performance, this small change may suggest that adolescents require longer training durations than adults for optimal adaptations. Kristensen et al. also report that resistance training for maximal speed should be performed at lighter loads to ensure that movement velocity remains close to that of an unloaded sprint [49].

The effects of training interventions on kinematic measures of the step cycle elucidate the intrinsic mechanisms underlying changes in sprint performance. All resisted groups showed significant increases in step length (1.62–4.45%) across the acceleration phase (0–30 m), while improvements in step frequency (3.65–7.32%) were mainly evident between 10 and 20 m. The more pronounced effect on step length is consistent with previous studies [5, 13, 34, 50]⁠. In contrast, Clark et al. [51] reported that attributing the enhanced athletic performance following resistance sled sprint training primarily to increased step frequency rather than step length. This may be attributed to their use of a lower load (about 10% BM) in maximum speed and the fact that their subjects were adult field hockey players rather than adolescent sprinters. Under sled sprint conditions, athletes must overcome external resistance, which initially reduces stride speed and leads to shorter step length [13, 43]. However, this resistance stimulates neuromuscular adaptations that enhance step length during non-resisted sprinting. For adolescent athletes, post the “window of trainability” for stride frequency development, training prioritizes enhancing stride length while maintaining stride frequency [52]. Specialized leg strength training enables athletes to achieve greater horizontal velocities within the same touchdown time, thereby increasing stride length without decreasing stride frequency [5, 36, 53]. In this study, we avoided the compensatory reduction in step length and acceleration of step frequency in adolescent sprinters due to resistance conditions. Coaches placed markers on the track to provide step length references and timed each sled sprint to ensure full sprint conditions, thereby maximizing step length consistency with unloaded sprinting. Although heavy loads alter running technique, they remain effective for improving acceleration.

We also observed significant reductions in flight time (0–20 m) in the 25% and 40% BM groups (4.17%-10.94% and 4,00%-10.77%, respectively) consistent with the findings of Stavridis et al. [23], who suggested that heavy-load resistance sprint training enhances athletes’ ability to generate greater off-ground horizontal velocity and explosive force, thereby reducing flight time. These improvements may stem from enhanced horizontal propulsion efficiency under such resistance loads [23], indicating potential gains in neuromuscular coordination and propulsion mechanics that contribute to overall sprint efficiency. However, a limitation is that neither GRF nor center of mass kinematics were measured in this study to directly confirm these mechanisms. Under resistance conditions, contact time increased and flight time decreased with higher loads, likely because heavier resistance shifted the landing support point behind the body’s center of mass, resulting in greater extension forces and prolonged contact time [12, 22, 51]. Combined with an increased forward trunk lean, this greater demand on the hip and leg extensor muscles may, as previous research indicates, involve recruiting more motor units for muscle contraction [3, 32, 35, 54]. Long-term training strengthens these muscles, reducing contact time and increasing lower-limb movement velocity [8]. In contrast, the 50% BM group showed no significant change, possibly due to compensatory neuromuscular adjustments at heavier loads in adolescents, as reflected by the lack of improvement in contact time.

In acceleration, the trunk should be inclined forward to lower the center of gravity. A pronounced forward trunk requires athletes to possess strong postural control, thereby increasing the technical training demands on young athletes. Studies have demonstrated that training with medium to heavy loads (>30% BM) in sled sprint increases forward trunk lean [29, 32]. Our results showed significant improvement in trunk angle: 50% BM group improved from 0 to 30 m (up to 10.03%), the 40% BM group from 0 to 20 m (up to 6.99%). These results are consistent with previous reports that different loads of sled sprint training had distinct effects on trunk forward lean during acceleration [13, 42]. The 40% and 50% BM groups showed significant improvements in push-off angle at 15 m, while the 25% BM group improved at 25 m. These outcomes support the findings of Bentley et al. [16], who suggest that the decrease in push-off angle after sled running is due to the resistance effect, which causes a reduction in lower limb joint flexion, activating stronger extensor reflexes in the extensor muscles. A smaller push-off angle indicates increased lower limb power during the push-off phase and greater forward propulsion of the body. Furthermore, the shin angle (touch down) decreased at 5 m and 25 m in the 25% and 50% BM groups (6.52% and 2.44%, respectively). However, no consistent trends were observed for this parameter, which may require further extension of the training period or may have some effect on trainers of other levels or programs.

It should be noted that participants in the resisted groups not only participated in loaded sled sprint training but also in the non-resisted sprint training following sled sprint. This may contribute to the transfer of training effects, potentially enhancing sprint performance in non-resisted conditions [55]. Research has shown that training adaptations vary among populations with different training levels and periods, even at the same resistance load intensity [8, 21]. Resistance sled training during the preparation phase is more effective than the other training phases [42]. The participants in our study were adolescent sprinters with 3–4 years of sprint training experience. The results might differ if the subjects had only trained in loaded sled running and no other training. Therefore, the findings are more applicable to sprinters of similar age and training levels.

There are several limitations to this study. First, the lack of kinetic data collection restricts the interpretation of results, particularly the interpretation of kinematic changes following training. Second, the findings may be specific to loaded sled run training during the acceleration phase and may not apply to other forms of resistance sprint training or other phases of sprinting (e.g., maximal speed or speed endurance). Third, while body mass-based loading offers practical utility for coaches, it may not provide optimal individual stimulus. Critically, the greater work output required for heavier sleds likely created inter-group differences in training volume, potentially confounding effects attributed solely to loading intensity. Future studies should implement velocity-power profiling to individualize athlete-specific resistances. Additionally, the small to moderate sample size limits the generalizability of the findings, meaning they may not be fully representative of the broader population. Finally, the participants were professionally trained adolescent sprinters (regional level), so the results may not apply to recreational athletes of the same age group.

## Conclusions

After an 8-week sled sprint training program with three different loads in adolescent sprinters, we investigated the effects on 30 m and 60 m sprint performance and kinematic parameters during acceleration. The results demonstrated that the 25% BM sled load enhanced 60 m sprint performance, while the 40% and 50% BM load improved 30 m sprint performance. All loading conditions increased step length during the initial 30 m of sprint and step frequency at 10–20 m. Additionally, 40% and 50% BM training reduced trunk angle (toe-off) and push-off angle during the early acceleration phase (0–20 m), compared to 25% BM training. These findings highlight the differential effects of sled load magnitude on sprint performance and kinematics in adolescent sprinters.

### Practical application

This study provides valuable insights into the optimization of resistance sprint training programs for adolescent sprinters. Our results indicate that a sled load of 25% BM is effective in enhancing 60 m sprint performance, likely through improvements in step length and maintenance of step frequency. Conversely, higher loads (40% and 50% BM) are more beneficial for improving 30 m sprint performance, with significant enhancements in step length, trunk angle, and push-off angle during the acceleration phase. These adaptations are crucial for maximizing sprint efficiency and performance in shorter distances. For coaches and trainers, it is recommended that sled sprint training cycles be appropriately integrated into the training programs of adolescent sprinters and heavier loads resistance sprint training be employed to enhance acceleration performance. Future studies should explore the long-term effects of varied sled loads on sprint performance in adolescent athletes.

## Supplementary Information

Below is the link to the electronic supplementary material.

Supplementary Material 1

## Data Availability

The data that support the findings of this study are available on request from the corresponding author.

## Declarations

### Statement of adherence to CONSORT guidelines

Although our study does not qualify as a clinical trial under the WHO definition, we have reviewed our manuscript in accordance with the CONSORT guidelines to ensure comprehensive and transparent reporting. We have made appropriate adjustments and have completed a CONSORT checklist, which is included as an additional file in our submission.

## Abbreviations

BM: Body mass
NRS: Non-resisted group
GRF: Ground reaction force
VDec: Velocity decrement
